# Increased colorectal cancer incidence in Iran: a systematic review and meta-analysis

**DOI:** 10.1186/s12889-015-2342-9

**Published:** 2015-10-01

**Authors:** Roya Dolatkhah, Mohammad Hossein Somi, Iraj Asvadi Kermani, Morteza Ghojazadeh, Mohamad Asghari Jafarabadi, Faris Farassati, Saeed Dastgiri

**Affiliations:** Hematology and Oncology Research Center, Tabriz University of Medical Sciences, 51666114731 Tabriz, Iran; Liver and Gastrointestinal Diseases Research Center, Tabriz University of Medical Sciences, Tabriz, Iran; Road Traffic Injury Research Center, Tabriz University of Medical Sciences, Tabriz, Iran; The University of Kansas Medical School-Molecular Medicine Laboratory, Kansas City, KS USA

**Keywords:** Epidemiology, Measurement, Rate, Standardization, Colorectal cancer, Registry

## Abstract

**Background:**

Colorectal cancer is the third most common cancer in Iran. The increasing trend of colorectal cancer incidence in Iran and the close relationship with the geographical location are the underlying reasons for this study.

**Methods:**

Data source: Eleven databases, including MEDLINE, EMBASE, SCOPUS, and four other databases, for articles in Persian were searched from April 2014 to October 2014. Additional data were obtained from an online survey of the Central Library of Tabriz Faculty of Medicine. Study eligibility criteria: In this systematic review and meta-analysis, we included studies reporting different measures of incidence, age-standardized incidence rates, and crude incidence rates. All rates (per 100,000 person-years) were standardized to the world standard population. Study appraisal and synthesis methods: A preliminary review of the title and abstracts of these articles was used to exclude any that were clearly irrelevant. The full text review determined whether the article was relevant to our topic. All the potentially relevant manuscripts were reviewed by two other investigators (S.D., M.G.). A total of 39 studies (10 Persian and 29 English articles) from different provinces and diverse areas of Iran, were analyzed in this study using comprehensive meta-analysis software. For accuracy studies, we used estimated rates for males and females with 95 % confidence intervals.

**Results:**

Age-standardized incidence rates were obtained based on the random effects model and were 8.16 (95 % CI: 6.64 to 9.68) and 6.17 (95 % CI: 5.01 to 7.32) for males and females, respectively. The random crude rates were 5.58 (95 % CI: 4.22 to 6.94) for males and 4.01 (95 % CI: 3.06 to 4.97) for females.

**Conclusions:**

Colorectal cancer incidence rates rise due to individual and environmental risk factors as well as improvement in the registry system and increase in access to health services. A more executed organized and structured system for collecting cancer data, in all cities and rural areas of the country, is an essential priority.

## Background

Cancer is one of the major global health problems, and its importance is on the rise in Iran as well. Cancer is the third most common mortality factor, and the second largest group of chronic non-communicable diseases in Iran. Cancer of the colon and rectum is one of the leading causes of cancer-related deaths in the Western countries, and colorectal cancer (CRC) is the third most common cause of cancer death in the world [1]. Colorectal cancer is the third most common cancer in Iran. The incidence of CRC significantly varies depending on the geographical regions. It varies at least 10 times in different regions of the world and has a significant time trend [2–4]. Furthermore, there is a significant difference in the incidence and mortality rates between different racial and ethnic groups [2]. While the annual incidence rate of CRC is 30–50/100,000 individuals in North America and Europe, it is about 37/100,000 in most Middle-Eastern countries. Cultural changes in the third-world countries have led to a rapid increase in the incidence of CRC in these countries [5, 6].

The National Cancer Registry Program in Iran (INCRS) was started in 2000, and its results have provided an important source of information for cancer studies in the country. This is pathology-based program with >85 % of the expected number of cancers being registered. According to the information obtained from this source, there is a significant difference in the incidence rates of CRC in different parts of Iran. According to the annual report of INCRS, CRC is the fourth common cancer in men after stomach, bladder, and prostate cancer and the second among women after breast cancer (besides melanoma) [7–10].

Recent studies have shown a rapid rise in the incidence of colorectal cancer in Iran [11, 12]. In a study done about 35 years ago in Iran, the estimated annual incidence rate of CRC was 1.5–5.5/100,000 individuals [1, 13]. A research study conducted in 2008 reported an age-standardized incidence rates (ASR) of 7–8 (per 100,000) for both males and females and further stated that 5000 new cases of CRC (7 per 100,000 individuals) are annually reported from Iran [1]. Of these 5000 cases, 2262 die annually because of CRC [3, 8, 9]. This is higher than that of previous reports in Iran, while it is closer to the statistics reported from other Middle East areas, and less than the rates in western countries [8, 14] . A recent study conducted in the North West of Iran in 2014 reported that the annual ASR were estimated to be 11.2 and 8.93 in men and women, respectively, and the respective crude rates (CR) were 11.5 and 9.22 [12, 15].

As compared to western countries, the incidence of colorectal cancer in Iran is low, but it seems to have increased significantly during the past decade [11, 12, 16]. There is a wide geographical variation in the incidence of cancers within the 31 different provinces in Iran. In addition, there are significant variations in the infrastructure and capacities of these provinces. A close relationship between cancer and the geographical location is also the underlying reason for this emerging trend, and the increased colorectal cancer incidence in geographically diverse areas of Iran [10]. The rising incidence and mortality rates of colorectal cancer demand a systematic approach toward a specific and national research program in Iran. Statistical data should be accurately and sufficiently updated periodically to identify the trends in the incidence, prevalence, and mortality of colorectal cancer in Iran. The aim of this systematic review and meta-analysis, was to report and analyze the different measures of incidence, age-standardized incidence rates and crude incidence rates of colorectal cancer from different areas of Iran.

## Methods

### Protocol and registration

This systematic review and meta-analysis adhere to the PRISMA guideline. As this study did not include any clinical trials nor compare between groups, we did not register it in the Cochrane library.

### Eligibility criteria

A preliminary review of the title and abstracts of articles was performed to exclude any irrelevant evidence. Full-text review was performed to determine whether the article is relevant to the study topic. All the potentially relevant manuscripts were reviewed by two other investigators (S.D., M.G.).

Abstracts or any unpublished studies such as thesis and reports were not considered in the analysis. Both English and Persian language articles were included in this study.

### Information sources

The databases PubMed and PMC, MEDLINE, EMBASE, SCOPUS, and Google Scholar were searched for articles in English and SID, Magiran, Medlib, and Irandoc for articles in Persian from April 2014 to October 2014. The reference lists of the obtained articles were searched to identify any additional relevant articles on the same topic. If the results of the study did not report ASRs and/or CRs in the primary article, we tried to contact the author for the information by email. In some cases, due to old date of the study and inaccessibility to electronic files, we requested the journals’ editorial office to send us the paper files via Fax.

### Search

For retrieving the published papers to identify the studies of interest, we conducted a computerized literature search. We used medical subject headings to identify all the relevant articles. The search terms were as follows: Colorectal Cancer (colorectal OR colorectum OR colon OR rectum OR bowel), Incidence Rate (Incidence OR age standardized rate OR ASR OR Crude Incidence Rate), Iran, Epidemiologic study, Characteristics, and Distribution.

### Study selection

Our selection criteria were any epidemiologic, analytic, descriptive, clinical, cross-sectional, and population-based studies including information about age, gender, and subsite of CRC in the different provinces of Iran. We included any results from January 2000 to October 2014.

Inclusion was not otherwise restricted by study size or language. Studies in which gender was not applied in the measures of incidence rate were excluded from the meta- analysis. We included the studies in which the overall incidence rates for colon and rectum cancers were reported.

### Data collection process

We also included the studies reporting different measures of incidence, age-standardized incidence rate, and crude incidence rates in this meta-analysis. Two reviewers (R.D., S.D.) abstracted the data to a predefined form. After computing the rate estimate for each study, we created a more complex model.

### Data items

The following data were collected from each study: The first author’s last name, publication date, and the province of origin; study cases and target population; duration of the study; characteristics of the study subjects (distribution according to age and gender); age-standardized incidence rate and crude incidence rate; anatomical subsite: cancers of the cecum, appendix, ascending colon, hepatic flexure of colon, and transverse colon were categorized as Right Side Tumors, and cancers of splenic flexure of the colon, descending colon, and sigmoid colon were categorized as Left Side Tumors. Cancers of the rectum and rectosigmoid were categorized as Rectal Cancers.

### Risk of bias in individual studies

Our data sources were primarily based on the Iranian National Cancer Registry System reports with a coverage rate of more than 85 % of cancer cases in the country [10]. The primary outcome was therefore identical in all the studies. Three studies were excluded due to the lack of sufficient data.

### Summary measures

The actual risks of cancers are better described by the age-standardized rate and the overall number of cancer cases each year, or the crude incidence rate. Therefore, the terms used to determine the outcome were Age-Standardized Incidence Rate (ASR) and/or Crude Incidence Rate (CR) of Colorectal Cancer. All ASRs and CRs per 100,000 person-years were obtained and standardized to the world standard population in all of the studies. If more than one ASR and/or CR was reported in a study, we used the latest data in the meta-analysis. ASRs were not reported in 11 studies, for which we calculated the ASR by direct standardization method using world standard population, in the different age and sex groups per 100,000 population [17]. The target population of each province was obtained from the recent data of the general census statistics of Iran [18].

### Synthesis of results

All the analyses were performed using the Comprehensive Meta-Analysis software, version 2.2 (Biostat, Englewood, New Jersey). Random effects models were used to calculate the ASR and CR incidence rates with 95 % confidence intervals; *P* < 0.05 was considered to be statistically significant. Forest plots were made for males and females separately for ASR and CR. Heterogeneity across the studies was assessed with the Cochran’s Q statistic-value, I^2^ Index, and degree of freedom (df), and *P* < 0.10 was considered to be statistically significant. The following thresholds were considered for the interpretation of I^2^ Index: 0 to 40 %: might not be important, 30 to 60 %: may present moderate heterogeneity, 50 to 90 %: may present substantial heterogeneity, and 70 to 100 %: considerable heterogeneity [19].

### Risk of bias across studies

Although the results of the random effects models did not show a considerably significant difference, we used the random effect model for minimizing the risk of bias across the studies [20, 21].

### Consent statement

As this is a systematic review and meta-analysis, we did not include any humans and/or animals. This study was approved by the Ethics Committee of Tabriz University of Medical Sciences.

## Results

### Study selection

**Fig. 1:**
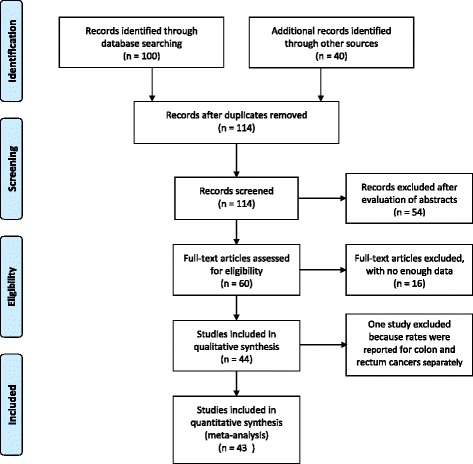
Flow Diagram of study selection process

Most of the data were retrieved from the Iranian Cancer Registry System, which collects the epidemiologic and clinical data of all newly diagnosed cancer cases. After excluding 26 duplicate studies, we included 39 studies, comprising 10 Persian and 29 English articles, conducted in different provinces and areas of the country, published from 2010 to 2014. Figure 1 shows the flow chart of the searches done for identifying the published and reported cases included in this study.

### Study characteristics

A total of 44 studies were included in this analysis. Among these, 33 studies reported age-standardized incidence rates and 24 studies reported crude incidence rates for both males and females. In addition, 8 studies reported the ASR results and the overall rates for both men and women and 16 studies summarized the ASRs as an overall rate. Moreover, 24 studies reported the overall ASRs, in which gender was not considered in the measures of incidence rates. These studies did not meet the inclusion criteria of the study, and we did not include them in the meta-analysis reports. The characteristics of the included studies are summarized in Table 1. All the estimated rates in whole parts of the study have been reported per 100,000 persons, and so the repetitions were ignored.

**Table 1:** Summary of studies for colorectal cancer rates (per 100,000) in Iran

First Author’s Surname, Year, Reference	Province of population studied	Duration of study	Study cases	Target population	Mean age (±SD)	Cases ≤40 year	ASR/100,000	CR/100,000	Gender Male/Female %	Anatomical subsite
Somi 2014 [15]	East Azerbaijan	2007–2011	366	3724620	59.4 (±14.7)	NP	M:11.2	M:11.5	55/45	NP
							F:8.93	F:9.22		
Rouhollahi 2014 [10]	Iran	2011–2013	7163	75149669	NP	NP	M:11.6	NP	NP	NP
							F:10.5			
Moradi Nejad 2014 [45]	Hormozgan	2011	23	1578183	M:56	NP	NP	M:1.1	55/45	NP
					F:51			F:1.2		
Vakili 2014 [46]	Yazd	2005–2009	NP	990818	M:60.07 (±19.49)	NP	2005–2009	NP	53.5/46.5	NP
							M: 6.1–9.9			
					F:54.93 (±18.03)		F:5.2–6.0			
Askarian 2014 [47]	Fars	2001–2009	1531	4.6 million	NP	NP	9Y	9Y	58/42	NP
							M:63.36	M:44.04		
							F:48.15	F:32.81		
Amoori 2014 [48]	Khouzestan	2004–2008	NP	4531720	NP	NP	M:11.6	NP	NP	NP
							F:10			
							Overall:10.8			
Fateh 2013 [49]	Shahroud	2000–2010	NP	234825	61.8 (±4.4)	NP	10Y	10Y	53.3/46.7	NP
							M:114.8	M:98.9		
							F:95.4	F:83.9		
							Overall:105.2	Overall:91.6		
Rohani-Rasaf 2013 [23]	Tehran	2008	NP	13422366	NP	NP	M:16.4	NP	NP	NP
							F:12.2			
Talaiezadeh 2013 [50]	Khuzestan	2002–2009	1102	4,531,720	52.91 (±20.95)	NP	M:6.32	M:4.32	51.4/48.6	NP
							F:5.72	F:3.45		
Abdifard 2013 [6]	West of Iran:	2000–2005	762	1:1945227	NP	NP	2000–2005	NP	NP	NP
	1. Kermanshah			2:1493645			1:07–6.2			
	2. Kordestan			3:557599			2:2.7–4.1			
	3. Ilam			4:175826			3:0.0–2.1			
	4. Hamedan			Overall:575739			4:1.8–4.6			
							Overall:1.5–4.8			
Hosseinzadeh 2012 [51]	Isfahan	2009	125	4879312	58.38 (±11.64)	5.6 %	Overall :2.37	NP	53.8/47.2	NP
Roshandel 2012 [30]	Golestan	2004–2008	611	1640200	M:62 (±25)	NP	M: 12.4	M:8.34	56.4/43.6	NP
					F:53 (±25)		F: 9.5	F:6.54		
Safaee 2012 [16]	INCRS	2005–2009	19617	70166948	58.9 (±15.4)	11 %	4Y	NP	56/44	Colon: 62 %
							M:39.96			Rectal: 35.1 %
							F:36.16			Anus & Anal canal: 2.9 %
							Overall:38.0			
Najafi 2011 [52]	Kermanshah	1994–2007	665	2000000	NP	NP	1993–2007	NP	59/41	NP
							3.5–5.7			
							Overall:4.5			
Rohani-Rasaf 2011 [53]	Tehran	2007	509	7803883	NP	NP	M:13.418	M:7.324	NP	NP
							F:4.284	F:5.710		
							Overall:4.940	Overall:6.522		
Haghdoost 2011 [2]	Kerman	1991–2002	551	2.6 million	56.4	NP	12Y	12Y	49.9/50.1	NP
							M:50	M:21		
							F: 53	F:22		
Zendehdel 2010 [54]	Iran	2004–2006	NP	70166948	NP	NP	M:7.1	M:6.1	NP	NP
							F:6.5	F:5.4		
Koosha 2010 [33]	East Azerbaijan	2007	208	3603456	54.97 (±18)	NP	M:7.98	M: 6.3	57.2/42.8	NP
							F:5.67	F: 4.82		
							Overall:6.78	Overall:5.55		
Mashhadi 2010 [24]	Zahedan	2003–2006	94	2405742	51 (±19)	NP	4Y	NP	58/42	NP
							M: 41			
							F: 53			
Keshtkar 2009 [55]	Golestan	2004–2005	47	1617087	52.2 (±13.8)	10.6 %	Overall:6.26	NP	NP	NP
Somi 2009 [14]	East Azerbaijan	2006–2007	337	3603456	M:54.53 (±14.21)	NP	M:11.57	M:10.2	55.4/44.6	NP
					F:6.91 (±14.41)		F:9.73	F:8.50		
Babaei 2009 [56]	Ardabil	2004–2006	241	1,228,155	59.8 (±17.8)	NP	M:9.6	M:7.6	59.75/40.24	NP
							F:7.4	F:5.3		
Ganji 2009 [57]	Iran	2000–2004	1198	63741000	NP	NP	M: 8.3	NP	53/47	NP
							F: 6.5			
							Overall: 7.4			
Abdollahian 2009 [58]	Lorestan	1999–2003	39	1754243	NP	0 %	8.94	NP	56.4/43.6	NP
Mousavi 2008 [59]	Iran	2005–2006	4056	10629166	NP	NP	M: 8.19	M:6.37	55.6/44.4	NP
							F: 7.56	F:5.36		
							Overall: 8			
Mehrabani 2008 [60]	Fars	1990–2005	197	4336878	NP	NP	M: 3.26	M:1.92	56.85/43.15	NP
							F:2.41	F:1.51		
Fakheri 2008 [3]	Mazandaran	Oct1999–Feb2008	296	2922432	52.6 (±15.24)	33.5 %(98)	8Y	NP	51.35/48.65	RS: 40.2 %
							Overall :10.94			LS: 18.58 %
										Rectal: 42.7 %
Salari 2007 [61]	Yazd	1992–1999	191	990818	NP	7.4 %	Overall:5.34	NP	55.5/44.5	RS: 16.2 %
										LS:35.1 %
										Rectal:32.5 %
Sadjadi 2007 [38]	Kerman	1996–2000	378	2004328	M:53.1 (±21.4)	NP	M: 5.9	M:3.9	52.64/47.36	NP
					F:49.5 (±19.3)		F:5.9	F:3.6		
Semnani 2006 [31]	Golestan	2006	139	1640200	NP	NP	M:16,	M:10.2,	60.43/39.57	NP
							F:9.2,	F:6.8		
							Overall:12.6	Overall:8.5		
Bafandeh 2006 [34]	East Azerbaijan-Tabriz	1992–2005	143	3603456	53.9 (±13.3)	13.3 %	Overall:10.44	NP	62.2/37.8	RS: 18.2 %
										LS:39.1 %
										Rectal: 42.6 %
Somi 2006 [35]	East Azerbaijan	1999–2004	NP	3,500,000	M:63.3 (±12.8)	NP	M:6.7	M:6.0	NP	NP
					F:59.5 (±13.4)		F:5.2	F:4.8		
Ansari 2006 [1]	Gilan	1996–2000	2055	9.5 million	NP	17 %	M:1.64	NP	54.9/44.0	NP
	Mazandaran						F:1.4			
	Golestan									
	Ardabil									
	Kerman									
Pahlavan 2005 [22]	1. Ardabil	1996–2000	1628	1.1168011	NP	NP	1.M:7.86	1.M:5.0	57.4/42.5	NP
	2. Mazandaran			2.1740772			F:5.89	F:3.3		
	3. Golestan			3.1522468			2.M:9.9	2.M:8.9		
	4. Kerman			4.2004328			F:8.4	F:7.0		
							3.M:10.7	3.M:7.4		
							F:6.5	F:4.2		
							4.M:5.9	4.M:3.9		
							F:5.9	F:3.6		
Sadjadi 2005 [62]	Golestan	1996–2000	3641	6.4 million	NP	NP	M: 8.3	NP	56.2/43.8	NP
	Mazandaran						F: 6.5			
	Kerman									
	Ardabil									
Babaei 2005 [63]	Semnan	1998–2001	NP	293000	59.41 (±19.08)	NP	M: 11.62	M:10.6	54/46	NP
							F: 10.52	F:8.4		
Jalali 2005 [64]	Iran	1981–2001	900	13422366	51.07 (±14.87)	26 %	Overall :7.82	NP	58/42	RS:12.7 %
										LS:22.91 %
										Rectal: 64.65 %
Emami 2005 [65]	Isfahan	1996–2003	1100	4559256	NP	15.2 %	7Y	NP	NP	RS:14.1
							Overall :24.95			LS:24.3 %
										Rectal: 61.6 %
Yazdanbod 2004 [66]	Ardabil	1996–1999	NP	1128864	NP	NP	M: 7.86	M:5.04	NP	NP
							F:5.89	F:3.33		
Nikshoar 2004 [67]	Iran	1992–2002	2107	13422366	NP	20.4 %	10 Y	NP	55.9/44.1	RS: 13.9 %
							M:20.84			LS:30.5 %
							F:17.56			Rectal:55.7 %
Sadjadi 2003 [68]	Ardabil	1996–1999	180	1128864	M:59.1 (±16.7)	NP	M: 7.9	M:5	60/40	NP
							F: 5.9	F:3.3		
					F:53.0 (±17.8)					
Sarmast 2002 [39]	Khuzestan	1992–1999	100	4274979	NP	30 %	Overall :3.53	NP	58/42	RS:13 %
										LS:42 %
										Rectal:45 %
Sotoudehmanesh 2002 [69]	Iran	1991–2000	380	13422366	53.9 (±15.6)	22.3 %	Overall :6	NP	60.3/39.7	NP
Molanaei 2000 [70]	Kurdistan	1995–1999	100	1440156	51.49	23 %	5Y	NP	61/39	NP
							Overall :8.82			

*NP* not presented, *Y* year, *RS* right site, *LS* left site, *ASR* age standardized incidence rate, *CR* crude incidence rate, *INCRS* Iranian annual of national cancer registration, *CGLD* research center of gastrointestinal and liver disease

### Risk of bias within studies

We did not check for any risk of bias within the studies, as the statistical analysis methods used for calculating the age-standardized incidence rate and crude rate measurements were identical in all the studies. We reported age-standardized (ASR) and crude incidence rates (CR) for men and women separately. If ASRs or CRs of a study were reported several times over the years, we took the latest rates [6, 22]. If a particular study reported the ASRs and/or CRs from different provinces in a report, we considered the reports in each province as a separate study in the analysis of data [6, 22].

### Results of individual studies

The highest ASR was reported from Tehran for males (16.4) [23] and from Zahedan for females (13.25) [24] during 2000–2014, where the overall ASRs obtained from the reports exceeded by a maximum rate of 12.60 and a minimum rate of 1.37. The CRs vary from 11.5 to 1.1 in men and 9.22 to 1.20 in women.

### Synthesis of results

The overall ASRs of colorectal cancer in both males and females were obtained from 21 studies. Based on the random effect model, the ASR was 5.92, 95 % CI (4.79 to 7.06), (*P* < 0.001). The Cochran’s test results indicate for a low heterogeneity of review (Q = 23.78, df = 20, I^2^ = 15.91, *P* = 0.252). We did not include these studies in our meta-analysis. Table 2 shows the results of statistical analysis of data in these studies, including the age-standardized incidence rate and crude Incidence rates split by gender.

**Table 2:** Summary Statistics for colorectal cancer rates (per 100,000) in Iran

Incidence rate	Gender	Effect sizes and 95 % confidence interval				Heterogeneity			
		Number studies	Point estimate	Lower limit	Upper limit	Q-value	df	I-squared	*P*-value
ASR	Male	29	8.16	6.64	9.68	68.18	28	57.69	<0.001
	Female	29	6.17	5.01	7.32	48.43	28	42.19	0.010
CR	Male	22	5.58	4.22	6.94	44.04	21	52.32	0.002
	Female	22	4.01	3.06	4.97	27.69	21	24.15	0.15

*ASR* age standard rate, *CR* crude rate

**Fig. 2:**
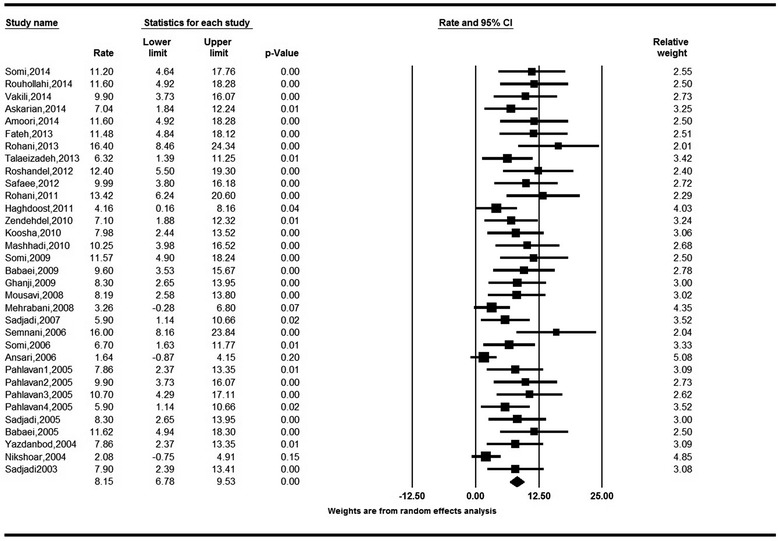
Forest Plot of random-effect meta-analysis for age standardized incidence rates in males in Iran, 95 % CI, weights are from random effects analysis

**Fig. 3:**
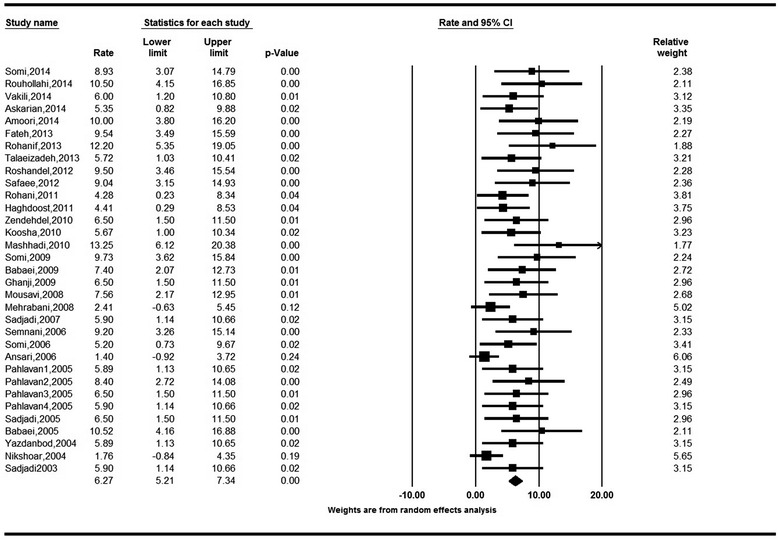
Forest Plot of random-effect meta-analysis for age standardized incidence rates in females in Iran, 95 % CI, weights are from random effects analysis

The age-standardized incidence rates (ASRs) of colorectal cancer obtained based on the random effect model were 8.16 (95 % CI: 6.64 to 9.68) for males and 6.17 (95 % CI: 5.01 to 7.32) for females. The Cochran’s test results indicate for a substantial heterogeneity of review in both males (Q = 68.18, df = 29, I^2^ = 57.69, *P* < 0.001) and females (Q = 48.43, df = 28, I^2^ = 42.19, *P* = 0.010). The heterogeneity of both analyses was in the range of moderate, as I^2^ was between 30 and 60 %. (Figs. 2 and 3).

**Fig. 4:**
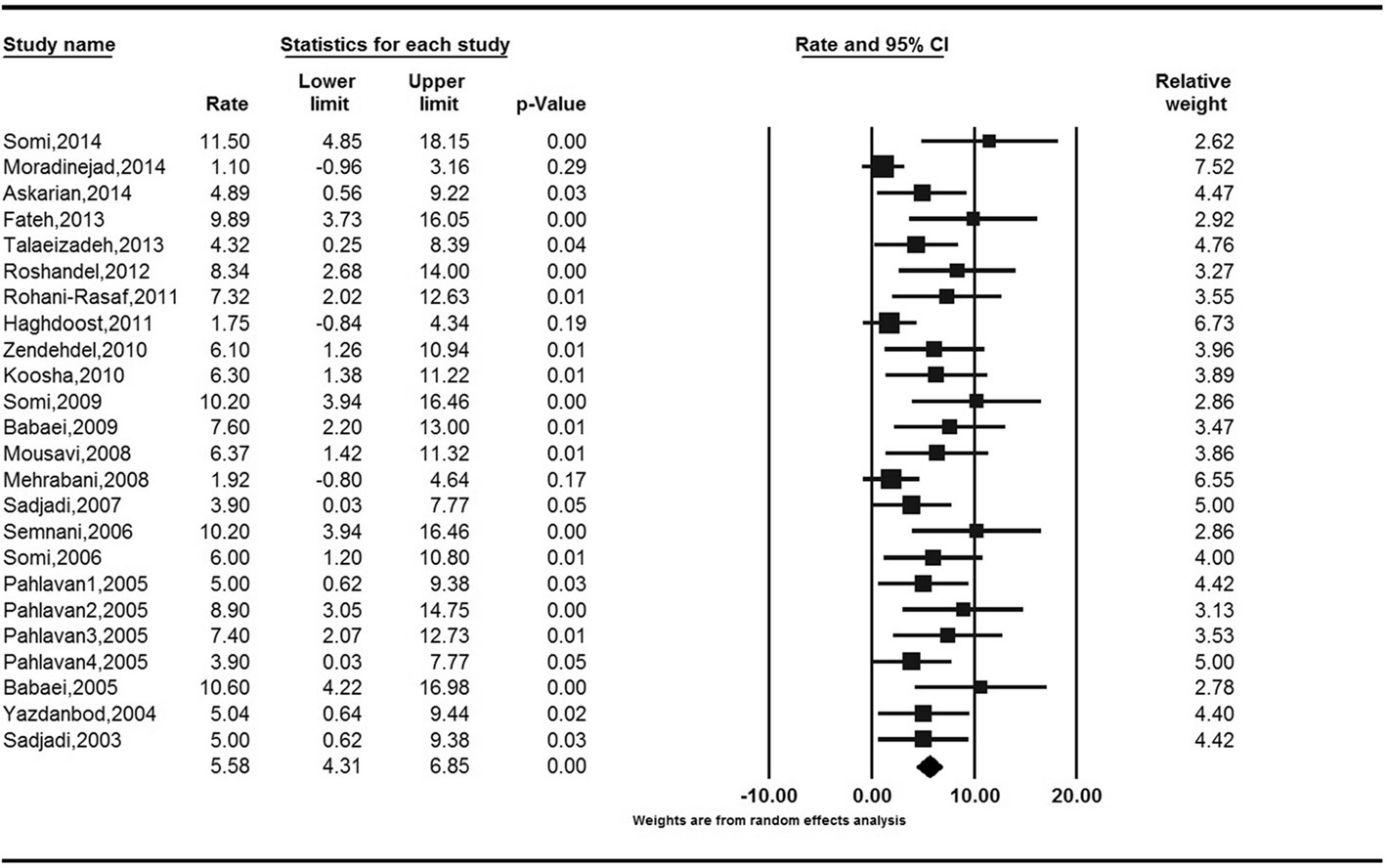
Forest Plot of random-effect meta-analysis for crude incidence rates in males in Iran, 95 % CI, weights are from random effects analysis

**Fig. 5:**
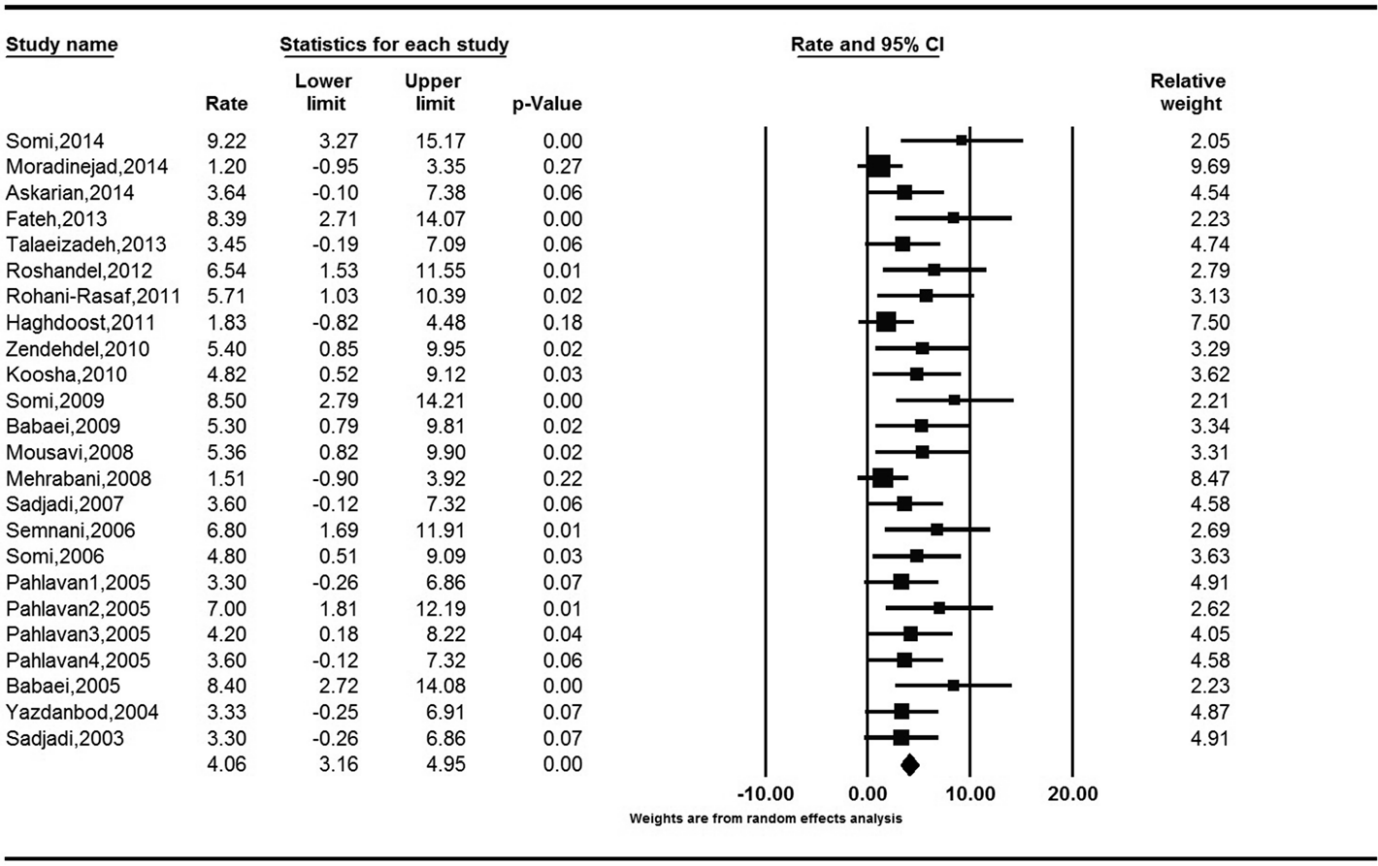
Forest Plot of random-effect meta-analysis for crude incidence rates in females in Iran, 95 % CI, weights are from random effects analysis

The crude incidence rates (CR) of colorectal cancer obtained based on the random model were 5.58 (95 % CI: 4.22 to 6.94) for males and 4.01 (95 % CI: 3.06 to 4.97) for females. The Cochran’s test results indicate for a moderate heterogeneity of review in both males (Q = 44.04, df = 21, I^2^ = 52.32, *P* = 0.004) and females (Q = 27.69, df = 21, I^2^ = 24.15, *P* = 0.15) (Figs. 4 and 5).

## Discussion

Iran is the second-largest nation in the Middle East. It is a large and diverse country, comprising several different religious and ethnic groups. It is divided into five main regions, with 31 provinces. Recently, Iran was ranked as an upper middle-income economy country by the World Bank. According to the GLOBOCAN 2012, colorectal cancer is the fourth most common cancer among men and the second among women in Iran [4]. The rising incidence and mortality rates of colorectal cancer demand a systematic approach toward a specific and national research program in Iran. We included in this meta-analysis the studies reporting different measures of incidence, age-standardized incidence rate (ASR), and crude incidence rate (CR). The data sources were primarily based on the Iranian National Cancer Registry System (INCRS) database results, in which more than 85 % of the expected numbers of cancers are being registered.

Globally, colorectal cancer is the second most common cancer in women and third in men. Although rates differ significantly in different parts of the world, incidence rates vary ten-fold in both genders worldwide. North America, Western Europe, Australia and New Zealand (with an ASR of 44.8 and 32.2 per 100,000 in males and females, respectively), have the highest estimated incidence rates. The lowest ASR was reported from Western Africa, with 4.5 and 3.8 per 100,000 for men and women, respectively [4].

Colorectal cancer is the third leading cause of cancer incidence and mortality in North America (North America includes Canada) in both genders and about 245,000 new cases occur annually in United States. The American Cancer Society(ACS) has reported the colorectal cancer ASR as 57.2 and 42.5/100,000 in men and women, respectively, during 2003–2007 [25]. According to the latest reports, these rates decreased to 50.0 and 37.8 per 100,000 in males and females, respectively, by 2011 [26]. The incidence and mortality rates have been decreasing slightly but steadily every year, and in March 2014, ACS released data showing that colon cancer incidence rates had dropped by 30 % in the last decade [27].

Colorectal cancer is the second most common cancer as reported in most European epidemiological studies. Its incidence rates have been steadily increasing from 1984 to 2007 according to some reports [28, 29]. Mistry et al. from United Kingdom have stated that although the ASRs for males and females were 56.2 and 37.3, respectively, in 2007, the incidence rate is projected to decrease by −7 % in males. Also, ASR for 2013 was projected to decrease to 52.3for men, but this theory is not applicable for women, according to this projection [28].

In this systematic review and meta-analysis, we included 39 studies from 2000 to 2014, from different provinces and areas of Iran. The northern part of the country is well-recognized to have the highest incidence of gastrointestinal tract cancers. The Golestan province, in northern Iran, is located at the western end of Asia.

Historically, esophageal cancer(EC) incidence is very high in this region [30]. Latest reports have shown a decrease in EC incidence rates, but an increasing trend in the incidence rates of CRC, and the highest overall ASR for colorectal cancer was reported from this province [31]. In Tehran city, the capital of Iran and the largest city in Iran and Western Asia, the incidence of CRC is higher than the WHO estimate for Iran. Colorectal cancer ASR has the highest rate (16.4) in males, and for women it is 12.2. It was higher than neighboring countries [12, 23, 32]. In an ecological study conducted in 2013 in all the 22 districts of Tehran, an interesting correlation between colorectal cancer incidence and different food groups, socioeconomic position scores, and other risk factors such as smoking was established. Colorectal cancer was found to be more common among the higher socioeconomic position groups, with a higher prevalence of risk factors, such as western dietary models, smoking, obesity, and low physical activity [23].

Several reports on the ASR and CR of colorectal cancer in East Azerbaijan province, in the North West of Iran, are also included in this review [8, 14, 15, 33–35]. It is located in Iranian Azerbaijan, bordering West Azerbaijan and Turkey, with Turkish culture. There is an increasing trend in CRC incidence, especially in females. According to our data, the highest CR for colorectal cancer was reported from this province, with 11.50 cases for males, and 9.22 cases for females. These rates are comparable with other regions of Iran and some neighboring countries, and should be considered in more comprehensive studies [12, 15]. Turkey is located in Western Asia with the portion of Eastern Thrace in southeastern Europe, bordering Iran from the East. Based on data retrieved from the Turkey Cancer Registry System, Yilmaz et al. estimated ASRs and CRs of all cancers from 2000. Colorectal cancer was the seventh most frequent cancer in both genders with lowest ASRs and CRs as compared to our results for the same Turkish cultural provinces of Iran. The overall ASR was about 7, and overall crude rate was 7.51 [36, 37]. In Turkey, cancer control programs aim to reinforce healthy nutritional habits by education and raising public awareness. Colorectal cancer screening programs were also introduced in more restricted parts of Turkey, which seem to be successful. Comprehensive screening programs could be useful for future planning of colorectal cancer control in Iran. Timely diagnosis and removal of adenomatous polyps may help in the prevention of this disease. Early diagnosis of local tumors may also improve the survival rate of patients. Educational programs on colorectal cancer screening may increase the overall knowledge about risk factors and screening modalities.

Several reports have shown that the ASRs and CRs of colorectal cancer in southern Iran are lower than in the north [2, 21, 38, 39]. The CRs in both genders were the lowest in Hormozgan province in the southeast of Iran, with 1.10 and 1.2, for males and females, respectively [21]. Nutritional habits (high consumption of vegetables and fruits), and genetic factors may play an important role. Also because of climatic conditions, date fruit is extensively grown and consumed in this part of Iran. Some studies suggested that dates have a strong antioxidant, which can potentially decrease the incidence and mortality rates of most gastrointestinal tract cancers.

Recently several reports revealed an increasing incidence and crude rates of colorectal cancer in Asian countries, which was comparable with other developed countries, especially in the younger population. Westernized lifestyle and diet, specifically high consumption of red and processed meat, obesity, and low physical activity have been established as the main cause of increasing risk of colorectal cancer in most Asian countries. However, how westernized diet increases the risk of cancer remains unknown, and could be due to both genetic and epigenetic polymorphisms [40].

While colorectal cancer was the fifth most common cancer among males, and the third in females in China [41], it was the fourth most frequent invasive tumors according to the data quality analysis on cancer incidence in six cancer registries in different parts of China, including urban and rural areas. The overall ASRs and CRs were respectively 14.61 and 21.33, in urban areas, and 11.09 and 12.43 in rural areas. This suggests other potential causes such as public education, promotion of healthy lifestyles and socioeconomic factors [42] . Hong Kong had a higher ASR and CR of colorectal cancer in recent years, and an increasing trend from 1983 to 2006. The overall crude rate was 57.1, and ASR was markedly higher in men than in women (68.2 and 47.1, respectively) [40].

In Lebanon, a small middle-income country on the Eastern Mediterranean shore, the ASRs for colon cancer in both genders are slightly higher than our and other results. Colon cancer was the fourth most common cancer among males, with ASR of 15.3, while it was the second in females with ASR of 14.1. The ASRs for rectal cancer were 4.2 and 4.8 in men and women, respectively. The higher ASRs for both colon and rectal cancers may be attributed to the highest elderly population in Lebanon among other Arab countries [43]. According to Saudi Cancer Registry in recent years, colorectal cancer was the most common cancer among men and the third in women. The overall ASR was 9.6, with 9.9 for men and 9.2 for women, which were similar to our rates in Iran [44].

### Limitation

The data sources for this study were primarily based on the INCRS database results, in which more than 85 % of the expected numbers of cancers are being registered. However, we did not have access to data from all cities and rural areas of the country.

## Conclusions

Colorectal cancer incidence rates rise due to individual and environmental risk factors as well as improvement in the registry system and increase in access to health services. Ageing population is often assumed to be the main factor driving an increase in colorectal cancer incidence and mortality rates; however the actual scenario is more complex. In developed countries, age-standardized mortality rates of cancer are now significantly decreasing in all age groups.

A more executed system for collecting cancer data, in all cities and rural areas of the country, is an essential priority. We emphasize monitoring the burden of colorectal cancer and increasing public awareness and attitudes toward the risk factors and prevention of cancer.
